# Identification and Functional Analysis of the *CgNAC043* Gene Involved in Lignin Synthesis from *Citrus* *grandis* “San Hong”

**DOI:** 10.3390/plants11030403

**Published:** 2022-01-31

**Authors:** Xiaoting Li, Naiyu Wang, Wenqin She, Zhixiong Guo, Heli Pan, Yuan Yu, Jianwen Ye, Dongming Pan, Tengfei Pan

**Affiliations:** 1 College of Horticulture, Fujian Agriculture and Forestry University, Fuzhou 350002, China; 2170305003@fafu.edu.cn (X.L.); 1200305016@fafu.edu.cn (N.W.); 000q020040@fafu.edu.cn (W.S.); gzhhs@fafu.edu.cn (Z.G.); panheli@fafu.edu.cn (H.P.); yyu@fafu.edu.cn (Y.Y.); 2 Agriculture and Rural Bureau of Pinghe County, Zhangzhou 363700, China; 1200305006@fafu.edu.cn

**Keywords:** NAC, MYB, gene expression, transcriptional activity, dual luciferase, pomelo

## Abstract

Overaccumulation of lignin (a physiological disorder known as granulation) often occurs during fruit ripening and postharvest storage in pomelo (*Citrus grandis*). It causes an unpleasant fruit texture and taste. Previous studies have shown that lignin metabolism is closely associated with the process of juice sacs granulation. At present, the underlying transcriptional regulatory mechanisms remain unclear. In this study, we identified and isolated a candidate NAC transcription factor, *CgNAC043*, that is involved in the regulation of lignin biosynthesis in *Citrus grandis*, which has homologs in *Arabidopsis* and other plants. We used the fruit juice sacs of ‘San hong’ as the material, the staining for lignin with HCl−phloroglucinol of fruit juice sacs became dark red from the various developmental stages at 172 to 212 days post anthesis (DPA). The RT-qPCR was used to analyze the gene expression of *CgNAC043* and its target gene *CgMYB46* in fruit sacs, it was found that the expression trend of *CgNAC043* was basically same as *CgMYB46*, which increased gradually and peaked at 212 DPA. The expression level of *CgNAC043* in juice sacs obtained away from the core was the lowest, while those near the core and granulated area were highly expressed. The transcriptional activation activity of *CgNAC043* and *CgMYB46* was analyzed by a yeast two-hybrid system, with only *CgNAC043* showing transcriptional activation activity in Y2H Gold yeast. A transformation vector, p1301- *CgNAC043*, was transformed into the mesocarp of ‘San hong’ by *Agrobacterium*-mediated transformation. Results showed that the expression of transcription factors *CgMYB58* and *CgMYB46* are all upregulated. Further experiments proved that *CgNAC043* not only can directly trans-activate the promoter of *CgMYB46* but also trans-activate the promoters for the lignin biosynthesis-related genes *CgCCoAOMT* and *CgC3H* by dual luciferase assay. We isolated the *CgNAC043* gene in pomelo and found *CgNAC043* regulates target genes conferring the regulation of juice sacs granulation.

## 1. Introduction

Pomelo (*Citrus grandis* (L.) Osbeck.) is a distinctive citrus fruit characterized by its thick peel, large size, and high vitamin C content [1]. “Sanhongmiyou” (San hong) is a new variety bred from the mutants of “Guanximiyou”. It is sour, sweet, delicious, and nutritious. In citrus fruit juice sacs, the overaccumulation of lignin (a physiological disorder known as granulation) often occurs [2,3]. Granulation of citrus fruits also occurs in “San hong”, especially during fruit ripening and post-harvest storage, affecting the commercial properties greatly [4]; it causes a “gritty” texture and dryness of the juice sacs, and reduces consumer acceptance of the fruits [2]. Lignin, which normally accumulates in secondary cell walls, is one of the most important components of the plant cell wall [5]. Previous evidence has shown that granulation due to excessive lignin accumulation can greatly damage fruit sensory quality during the post-harvest process [6]. 

The NAC (no apical meristem (NAM), *Arabidopsis* transcriptional activator (ATAF), and cup-shaped cotyledon (CUC)) family is one of the largest classes of plant-specific transcription factors (TFs) discovered in the past decade [7]. The NAM (no apical meristem) gene was first isolated from petunia in 1996. Subsequently, ATAF1/2 and CUC2 genes with similar effects were found in *Arabidopsis thaliana* by Aida [8]. There is a highly conserved DNA binding domain in the N-terminal region, whereas the C-terminal region of NAC proteins is a diversified activation domain [8,9]. 

MYB and NAC TFs play important roles in the transcription and regulation of lignin synthesis, as both transcriptional activators and repressors [10,11,12]. NAC TFs have been shown to participate in diverse biological processes, including the development of root and shoot apical meristems [13,14], organogenesis [15], hormone signaling [16,17,18], fruit ripening [19,20], responses to biotic and abiotic stresses [21,22,23,24], and leaf senescence in various plant species [25,26,27]. In addition, NAC TFs regulate the fiber biosynthesis and development of the secondary cell wall [28,29,30,31,32,33]. In dicot species, including *Arabidopsis*, the overexpression of *SND1* in *Arabidopsis* results in activation of the expression of secondary wall biosynthetic genes, leading to a massive deposition of the secondary walls [10]. Overexpression of *GhFSN1* in gossypium thickens the fibrage of the second cell wall [33]. *EjNAC1* is related to lignin synthesis and can activate the promoter that participates in the lignin synthesis pathway in loquat (*Eriobotrya japonica*) [34]. Overexpression of *BpNAC012* activates the expression of the secondary cell wall associated genes, resulting in ectopic deposition in the stem epidermis [35]. Previous findings have revealed that *AtMYB46*/*AtMYB83* is a direct target of *AtSND1* and is another key player in the transcriptional network involved in the regulation of secondary cell wall biosynthesis in *Arabidopsis* [36,37]. Overexpression of *AtMYB46* or *AtMYB83* up-regulates lignin synthesis-related genes, resulting in abnormal secondary cell wall accumulation [38]. In orange (*Citrus sinensis*), *CsMYB330* and *CsMYB85*, which are homologs of *AtMYB58*/*AtMYB63* and *AtMYB85*, respectively, have been reported to interact with *CsMYB308* through the binding of the *Cs4CL1* promoter to regulate juice sac granulation [2,39]. Subsequently, in pomelo, Shi [40] revealed the expression of *CgMYB58* led to lignin accumulation and the upregulation of 19 lignin biosynthetic genes. Among these, *CgPAL1*, *CgPAL2*, *Cg4CL1*, and *CgC3H* were directly modulated by *CgMYB58* through interaction with their promoter regions. In loquat, several MYB TFs are associated with lignin synthesis; *EjMYB1* and *EjMYB2* have been found to regulate lignin metabolism by binding to the AC element of the *Ej4CL1* promoter [41].

Zhong [42] reported that second wall NACs (SWNs) bind to a common cis-acting element, namely second cell wall binding element (SNBE), which is composed of an imperfect palindromic 19-bp consensus sequence, (T/A)NN(C/T)(T/C/G)TNNNNNNNA (A/C)GN(A/C/T)(A/T). Subsequently, the studies of EMSA and transactivation assays have demonstrated that representative SNBE sequences from the promoters of these target genes are bound and activated by not only *SND1* and *VND7*, but also by other SWNs, including *NST1/2* and *VND6* [42]. *OsSWNs* and *ZmSWNs* activate their target genes by directly binding to SNBE sites, leading to ectopic deposition of cellulose, xylan, and lignin [43]. 

The literature is rich in NAC TFs related to lignin metabolism, however, similar studies are lacking in citrus fruits juice sac lignification. In this report, we studied the functions of *CgNAC043* in the transcriptional control of secondary wall biosynthesis. We show that *CgNAC043* and *CgMYB46* are expressed in secondary wall-forming cells and they are functional orthologs of *NST1* and *AtMYB46*, respectively. When overexpressed, *CgNAC043* in pomelo is able to activate the expression of *CgMYB46* and *CgMYB58*. We further demonstrate that SNBEs are present in the promoters of *CgMYB46* and the genes related to the lignin synthesis pathway, and they could be directly activated by *CgNAC043*. Our results demonstrate that the secondary wall-associated NAC and MYB46 genes are key transcriptional switches regulating secondary wall biosynthesis in pomelo.

## 2. Results

### 2.1. Microscopic Observation of Lignin Determination of Lignin Juice Sacs

**Figure 1:**
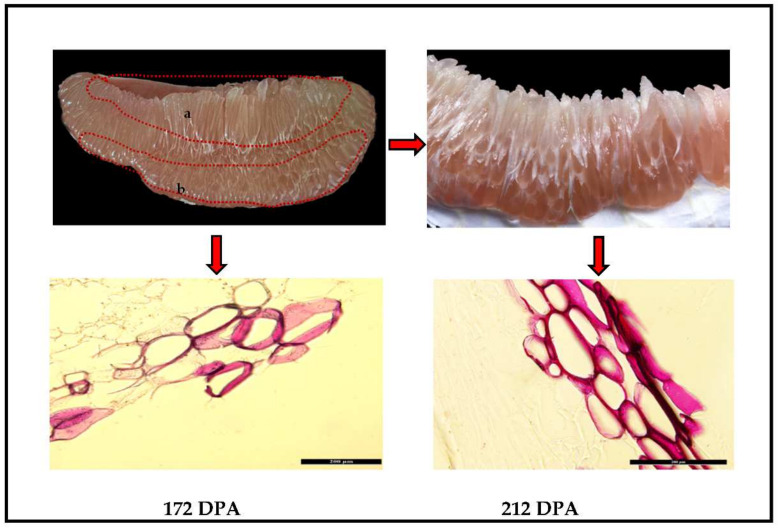
Morphology and staining of fruits juice sacs of “San hong” from 172 DPA to 212 DPA: (**a**) the tissues of near the core; (**b**) the tissues of far away from the core. Scale bar = 200 µm.

Representative images of morphological changes in pomelo fruits are presented in Figure 1. Juice sacs at later maturation (212 DPA) are stained darker red with the lignin specific stain HCl−phloroglucinol relative to the juice sacs at earlier maturation (172 DPA). We observed that juice sacs in “San hong” at 212 DPA exhibited the most severe lignification, indicating a relatively high level of lignin accumulation.

### 2.2. Identification of CgNAC043 and Sequence Alignment

The *CgNAC043* *(Cg5g000340.1)* cDNA was isolated from San hong, encoding a protein of 399 amino acids with a predicted molecular mass of 45.15 kDa and theoretical pI of 6.18. The total average hydrophilic coefficient was −0.867, indicating that this protein was hydrophilic. The instability coefficient of the protein was 57.62, indicating that the protein was unstable. 

**Figure 2:**
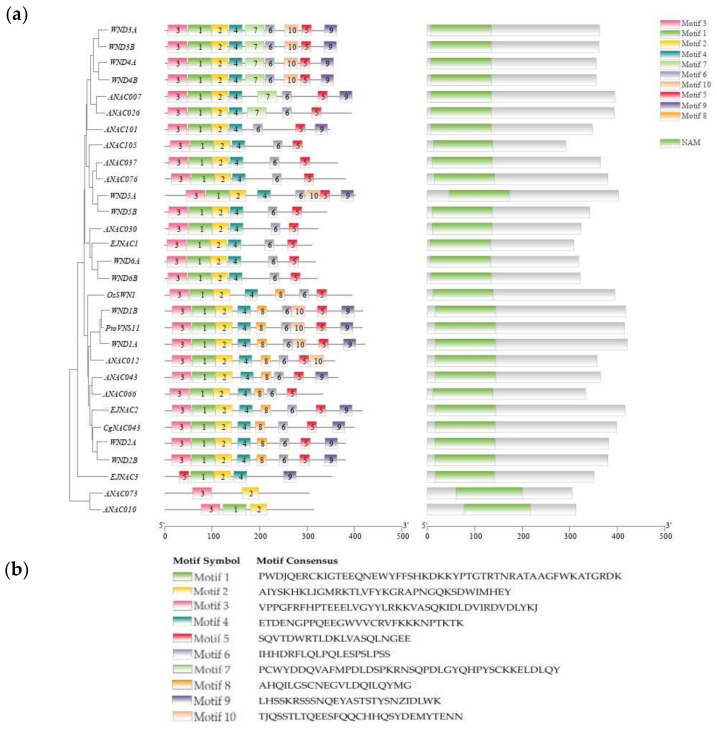
(**a**) Distribution of the conserved motifs and structural domain of NAC genes; (**b**) The sequences of the 10 motifs are exhibited at the bottom. *ANAC012* (AT1G32770.1), *ANAC073* (AT4G28500.1), *ANAC010* (AT1G28470.1), *ANAC043* (AT2G46770.1), *ANAC066* (AT3G61910.1), *ANAC037* (AT2G18060.1), *ANAC076* (AT4G36160.1), *ANAC105* (AT5G66300.1), *ANAC007* (AT1G12260.1), *ANAC026* (AT1G62700.1), *ANAC101* (AT5G62380.1), *ANAC030* (AT1G71930.1), *WND1A* (HQ215847.1), *WND1B* (HQ215848.1), *WND2A* (HQ215849.1), *WND2B* (HQ215850.1), *WND3A* (HQ215851.1), *WND3B* (HQ215852.1), *WND4A* (HQ215853.1), *WND4B* (HQ215854.1), *WND5A* (HQ215855.1), *WND5B* (HQ215856.1), *WND6A* (HQ215857.1), *WND6B* (HQ215858.1), *EJNAC1* (KJ919962.1), *EJNAC2* (KJ919963.1), *EJNAC3* (MG203936.1), *OsSWN1* (JN634070.1), and *PtoVNS11* (KU049786.1).

Multiple sequence alignments of the NAC proteins from *Citrus* *clementina*, *Citrus sinensis*, *Pistacia vera*, *Durio zibethinus*, *Gossypium hirsutum*, *Populus trichocarpa*, and *Citrus grandis* indicated that the *CgNAC043* protein shares 99.25%, 99.25%, 76.43%, 72.21%, 68.75%, and 65.84% identity with these species, respectively (Supplementary Figure S1). The structure of the domain showed that the *CgNAC043* protein contains a highly conserved NAM domain in the N-terminal portion. The conserved motif distribution displayed that motif3, motif2, and motif1 in the N-terminal region were highly conserved in all NAC proteins (Figure 2a). 

**Figure 3:**
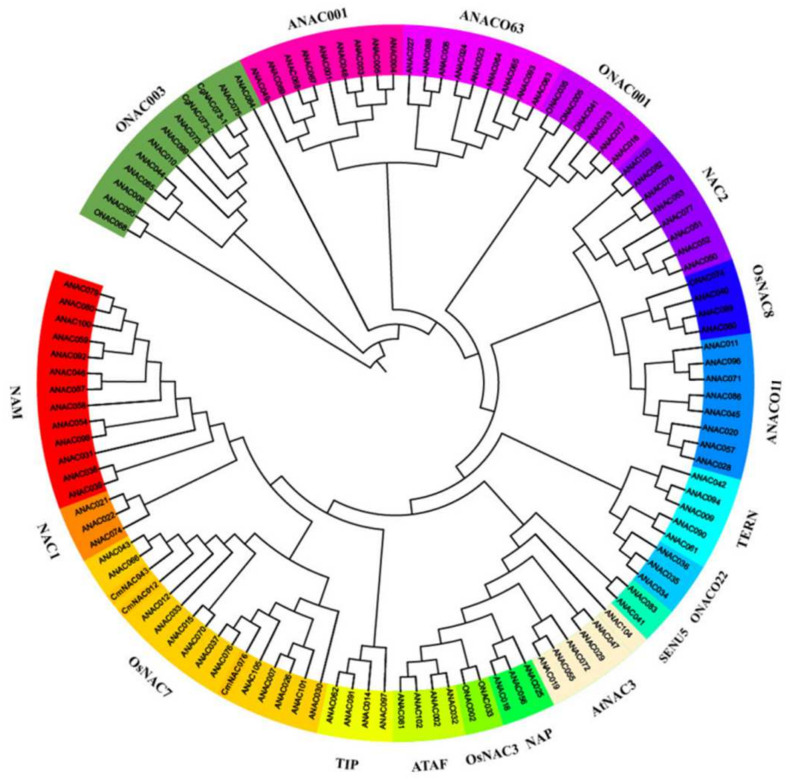
Phylogenetic analysis of NAC genes in *Arabidopsis thaliana*. The phylogenetic tree was constructed using the neighbor-joining method in the MEGA 6.0 program, and the bootstrap value was set to 1000. Each group is highlighted in a different color. Group I includes 14 sub-families, namely *ONAC022*, *TERN*, *SENU5*, *OsNAC7*, *OsNAC3*, *ATAF*, *AtNAC3*, *NAP*, *NAC2*, *ANAC011*, *TIP*, *OsNAC8*, *NAC1*, and *NAM*, while Group II includes four sub-families, namely *ONAC003*, *ANAC001*, *ANAC063*, and *ONAC001*.

Furthermore, to analyze the phylogenetic relationship of the secondary cell wall NAC proteins, an unrooted neighbor-joining (NJ) phylogenetic tree was constructed according to the full-length protein sequences in *Arabidopsis thaliana* (https://www.arabidopsis.org/, accessed on 18 November 2021). The results showed that *CgNAC043*, *ANAC043 (NST1)*, *ANAC066*, *ANAC012 (SND1)*, *ANAC043*, *ANAC033*, *ANAC015*, *ANAC070*, *ANAC037*, *ANAC076*, *ANAC0105*, *ANAC007*, *ANAC0101*, and *ANAC030* are located in the subfamily *OsNAC7*, suggesting that *CgNAC043* may be similar to these species in function (Figure 3). 

### 2.3. The Correlation Analysis of CgNACs and CgMYBs in the Process of Lignin Synthesis

**Figure 4:**
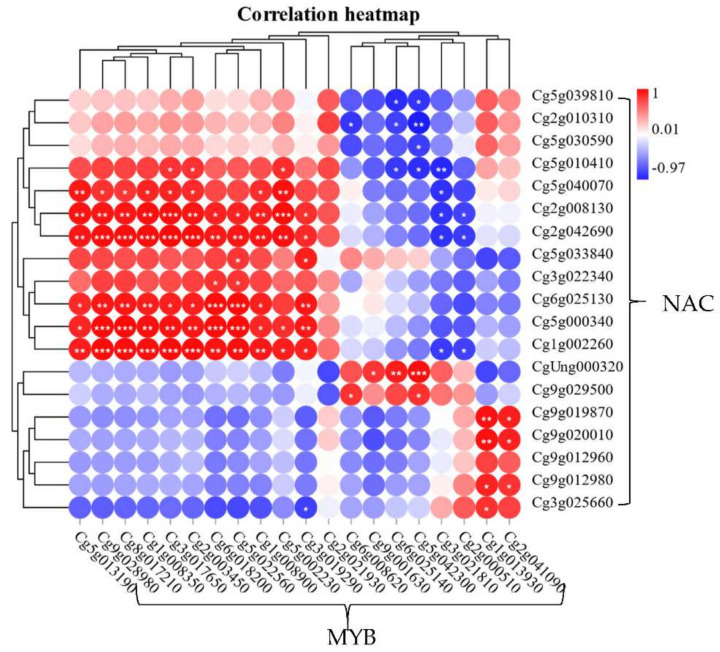
The correlation heatmap of *CgNACs* and *CgMYBs*. This heatmap is marked with different colors; scale bars represent different correlation coefficients. The FPKM values of *CgNACs* and *CgMYBs* in different tissues and different developmental stages of San hong were obtained from RNA-seq data of our lab. *: 0.01 < *p* < 0.05; **: 0.001 < *p* < 0.01; ***: *p* < 0.001.

We screened out two major types of transcription factors through RNA-seq data, namely *CgNACs* and *CgMYBs.* The FPKM values of these genes at 157 DPA, 180 DPA, and 212 DPA gradually increased with the synthesis of lignin (Supplementary Table S1). After further analysis of the correlation levels of these DEGs, we found that the correlation coefficient between *CgNAC043* (*Cg5g000340*) and *CgMYB46* (*Cg2g003450*) was 0.95, which is a highly positive correlation (*p* < 0.01) (Figure 4). This indicates that they may interact with genes of the lignin synthesis pathway and participate in the granulation process of San hong juice sacs.

### 2.4. Protein-Protein Interaction Analysis of CgNAC043

**Figure 5:**
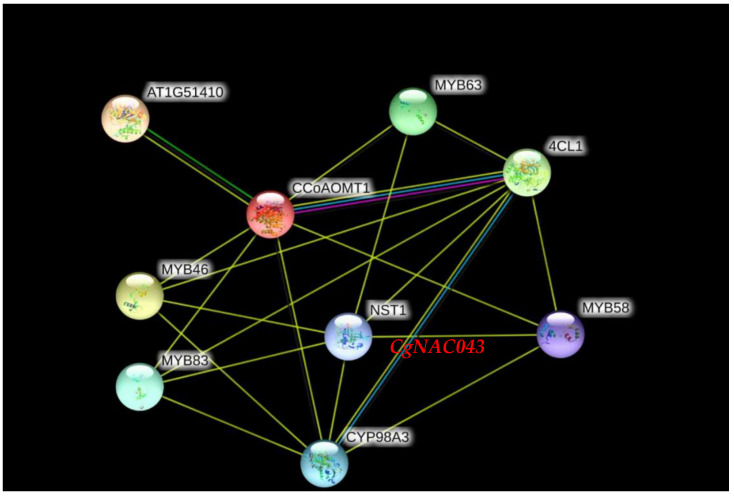
Interaction network of six proteins related to lignin biosynthesis on the basis of *Arabidopsis* orthologs. Medium confidence was set to 0.400.

Based on the *Arabidopsis* protein database, the protein–protein interactions of the six genes related to lignin biosynthesis were predicted using STRING software. *CgNAC043* was identified to be a homologous protein of *Arabidopsis NST1* (*AT2G46770.1*). *CgMYB46* was identified to be homologous protein of *Arabidopsis MYB46* (*AT5G12870.1*). *CgMYB58* was identified to be homologous protein of *Arabidopsis MYB58* (AT1G16490.1). *CgC3H*, *CgCCoAOMT*, and *Cg4CL* were identified to be homologous proteins of *Arabidopsis CYP98A3* (*AT2G40890.1*), *CCoAOMT1*(*AT4G34050.1*), and *4CL1* (*AT1G51680.1*), respectively. These genes were predicted to have the ability to interact with the *CgNAC043* protein (Figure 5).

### 2.5. Expression Patterns of CgNAC043 Gene

**Figure 6:**
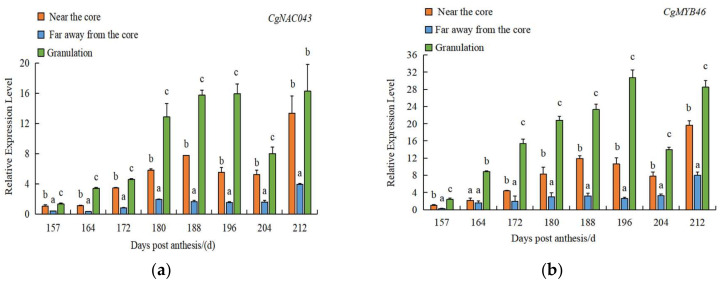

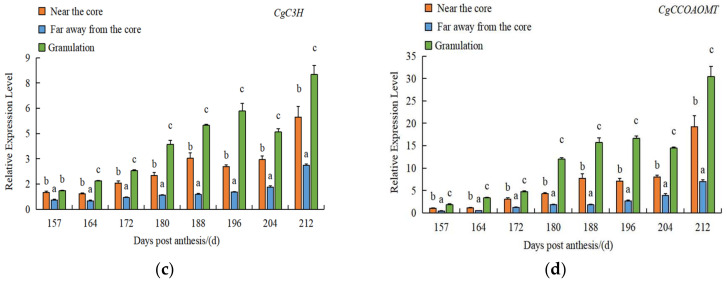
(**a**) RT-qPCR analysis of the expression level of *CgNAC043* in different parts of pomelo at 157–212 DPA; (**b**) RT-qPCR analysis of the expression level of *CgMYB46* in different parts of pomelo at 157–212 DPA; (**c**) RT-qPCR analysis of the expression level of *CgC3H* in different parts of pomelo at 157–212 DPA; (**d**) RT-qPCR analysis of the expression level of *CgCCoAOMT* in different parts of pomelo at 157–212 DPA. The expression level of near the core’s juice sacs at 157 DPA was used as a control (expression value = 1), the *β*-tublin gene was used as a reference gene for gene expression normalization. Significant difference between samples is indicated by different letters, which were found using the two-way ANOVA in the IBM SPSS Statistics 20. Error bars indicate SE (*n* = 3).

The expression of *CgNAC043* in the juice sac of San hong was analyzed to determine the regulatory role in the granulation of pomelo. The results showed that the expression level of *CgNAC043* in juice sacs obtained away from the core was the lowest, while those near the core and granulated area were highly expressed. The expression in granulation increased from 157 to 212 DPA, its expression reached the highest level at 212 DPA (Figure 6a). Moreover, *CgMYB46* (Figure 6b), *CgC3H* (Figure 6c), and *CgCCoAOMT* (Figure 6d) exhibited a similar expression pattern to that of *CgNAC043*, suggesting their possible involvement in secondary wall biosynthesis.

### 2.6. Transcriptional Activation of CgNAC043 and CgMYB46 in Yeast 

**Figure 7:**
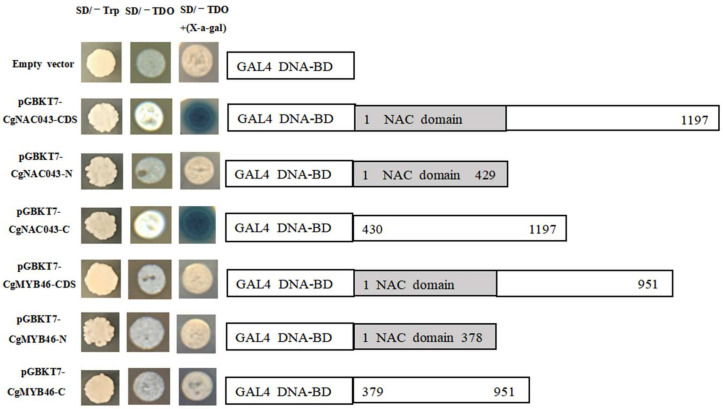
The full-length N-terminal and C-terminal sequences of CDS (coding sequence) of *CgNAC043* and *CgMYB46* to the GAL4 DNA-binding domain in pGBKT7 (BD) after transformation into Y2H gold yeast cells. The transformed cells were plated onto SD/–Trp (growth control), SD/–TDO, and SD/–TDO (+X-a-gal) medium.

The full-length *CgNAC043* and *CgMYB46* sequences and their truncated N-terminal forms (1–429 nt and 1–378 nt of *CgNAC043* and *CgMYB46*) and C-terminal (430–1197 nt and 379–951 nt of *CgNAC043* and *CgMYB46*) were inserted into pGBKT7-BD vector, generating fusion proteins with GAL4 BD. Yeast colonies containing the N-terminal region of *CgNAC043* failed to grow on SD/–TDO and SD/–TDO (+X-a-Gal). By contrast, yeast colonies containing the full-length and C-terminal region of *CgNAC043* not only grew on SD/–TDO, but also showed blue on SD/–TDO (+X-a-Gal), which indicated that the *CgNAC043* had a transcriptional activation activity and the activation motif was located in the C-terminal region. However, yeast colonies containing the N-terminal and C-terminal region of *CgMYB46* failed to grow on SD/–TDO and SD/–TDO (+X-a-Gal), indicating a lack of transcriptional activity of *CgMYB46* in Y2H gold yeast (Figure 7).

### 2.7. Transient Expression in Mesocarp

**Figure 8:**
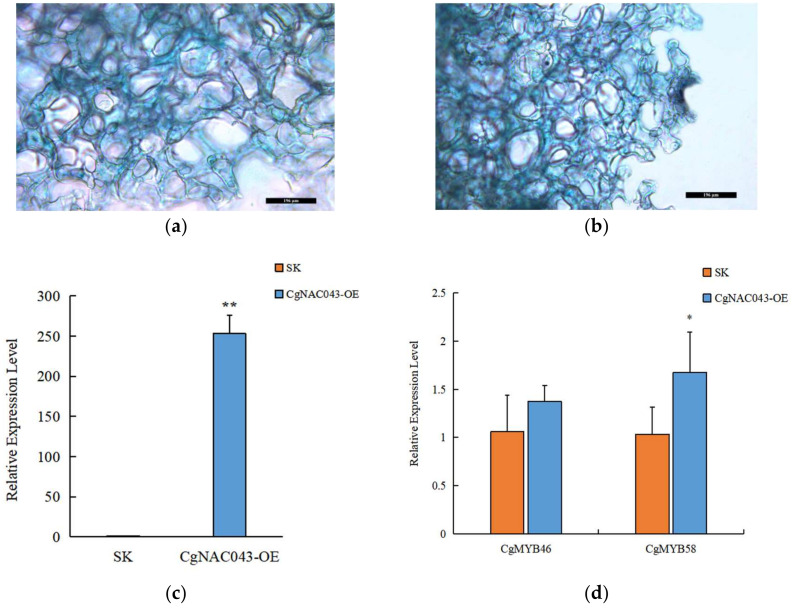
(**a**) Stained mesocarp of the SK control. (**b**) Stained the mesocarp of P1301-*CgNAC043*. Scale bar = 196 µm. (**c**) Expression levels of the *CgNAC043* gene in transiently overexpressing mesocarp tissue determined by RT-qPCR. (**d**) Transcription levels of *CgMYB46* and *CgMYB58* in transiently overexpressing mesocarp tissues determined by RT-qPCR; the *β*-tublin gene was used as a reference gene for gene expression normalization. Empty p1301 was used as a control (SK). Error bars indicate SE (*n* = 3). Student’s t-test: *: 0.01 < *p* < 0.05; **: 0.001 < *p* < 0.01.

To verify the biological function of *CgNAC043* in lignin synthesis, we transiently expressed *CgNAC043* in the mesocarp of pomelo fruits using *Agrobacterium* infiltration. The Gus staining of the mesocarp in the empty vector p1301 (SK) and p1301-*CgNAC043* are shown as blue (Figure 8a,b). The RT-qPCR analysis showed that the expression level of *CgNAC043* in transgenic plants was significantly higher than in the SK control plants (Figure 8c), indicating that the *CgNAC043* gene was successfully expressed. Further RT-qPCR analysis showed that the expression levels of *CgMYB46* and *CgMYB58* were upregulated in the transgenic mesocarp, which were 1.38- and 1.67-fold higher than those of the SK control, respectively (Figure 8d).

### 2.8. Trans-Activation by CgNAC043 of Promoters of Lignin Transcription Factors and Biosynthesis Genes

**Figure 9:**
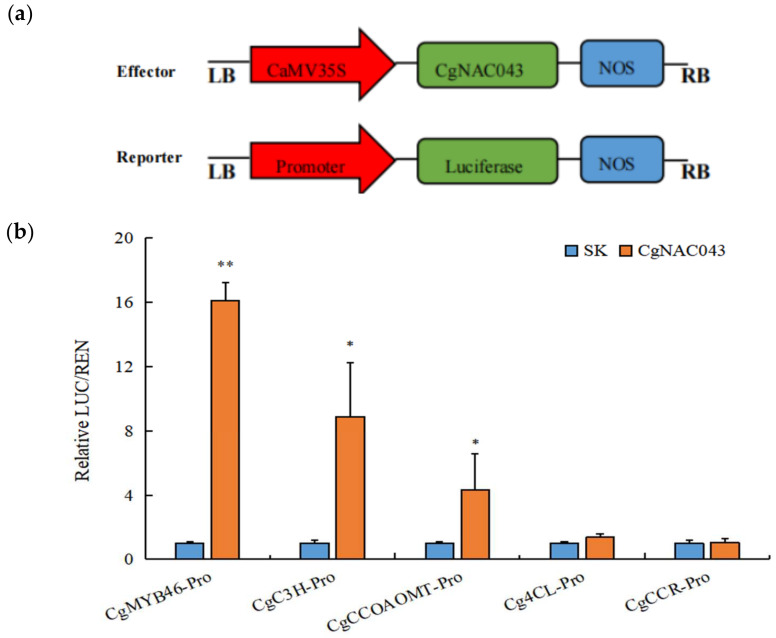
(**a**) The construct of effector and reporter. (**b**) *CgNAC043* on the promoters of lignin biosynthesis genes as determined by dual-luciferase assays. The ratio of LUC/REN fluorescence obtained with the empty vector 62sk plus the promoters was used as a control (SK) (set as 1). Error bars indicate SE from three replicates. Student’s *t*-test: *: 0.01 < *p* < 0.05; **: 0.001 < *p* < 0.01.

According to the results derived from RT-qPCR and transient expression, *CgNAC043* transcript levels are highly correlated with the lignification of the fruit juice sac. In order to further determine whether *CgNAC043* is directly involved in the lignification process of pomelo, we constructed effector and reporter fusion protein for transient transformation via *Agrobacterium* injection methodology (Figure 9a). The results indicated that *CgNAC043* could activate promoters of *CgMYB46* by approximately 16.1-fold. Furthermore, *CgNAC043* had effects on others promoters, such as *CgCCoAOMT* and *CgC3H*, by approximately 8.9- and 4.3-fold, respectively (Figure 9b). McCarthy [37] showed that *AtMYB46* was specifically expressed in fibers and ducts with secondary wall thickening, and its expression is directly regulated by *SND1* and its homologous genes, including *NST1*, *NST2*, *VND6*, and *VND7*. In this study, *CgNAC043* was involved in the lignification of juice sacs by regulating the lignin biosynthesis genes, such as *CgMYB46* and *CgC3H*, as well as other genes that may not have been studied. At the same time, this regulation may be related to the number of SNBEs elements involved in the lignin biosynthesis promoter (Table 1).

## 3. Discussion

Previous studies have shown that cell wall thickening and lignin deposition in fruit juice sacs occur during granulation [44]. The content of lignin, hemicellulose, and cellulose all increased in the cell wall of granulated juice sac [3]. A number of NAC TFs related to lignin metabolism have been widely reported, while their functions in citrus have never been researched. In this study, we isolated and identified a NAC gene *CgNAC043* from San hong. Sequence analysis based on alignment and phylogenetic tree construction indicates that *CgNAC043* is conserved with its orthologs, which have been associated with lignin biosynthesis in other plant species (Figure 2). Meanwhile, *CgNAC043* is adjacent to *PtrWND2A* and *PtrWND2B* in the phylogenetic tree, and is in the same branch as *ANAC043* and *EjNAC2.* Previous studies have shown that the overexpression of *PtrWNDs* in *Arabidopsis snd1nst1* double mutants can effectively supplement for defects of the fiber secondary wall and activated the whole secondary wall biosynthesis process. Overexpressed *PtrWND2B* and *PtrWND6B* can induce the expression of secondary wall-related TFs and its biosynthesis genes [45]. In loquat, *EjNAC1* can activate the promoters of the loquat lignin synthesis gene directly [34]. Therefore, we surmise that *CgNAC043* may regulate lignin biosynthesis during fruit juice sac granulation in pomelo. 

*CgNAC043* expression was found to increase significantly during the progression of juice sac granulation, which is consistent with the expression of *CgMYB46* (Figure 6). The results showed that the *CgNAC043* had the lowest expression level in the tissues far away from the core, and the highest expression level in the tissues near the core and granulated. Juice sac granulation is usually prone to the tissues near the core, and then gradually develops to the tissues away from the core [44]. At the same time, we found the staining of fruit juice sacs in San Hong became progressively stronger (Figure 1). So, we speculated that *CgNAC043* may accelerate granulation during this process, which leads to the high level of lignin accumulation in juice sacs.

Some members in MYB and NAC TFs families are involved in the regulation of lignin biosynthesis in *Arabidopsis*. *AtMYB85*, *AtMYB58*, *AtMYB63*, *AtMYB46*, *NST1*, *NST2*, *SND1*, and *SND2* are involved in the regulation of secondary cell wall biosynthesis [10,37,46,47,48]. Shi showed that the transient expression of *CgMYB58* led to an increase in the lignin content in the pomelo fruit mesocarp, whereas its stable overexpression significantly promoted lignin accumulation and upregulated 19 lignin biosynthetic genes [40]. Zhong demonstrated that the *AtMYB46* transcription factor is a direct target of *SND1*, which is a key transcriptional activator regulating the developmental program of secondary wall biosynthesis [36]. In this study, we transferred the *CgNAC043* gene into the mesocarp of pomelo and found that it could up-regulate the expression of *CgMYB46* and *CgMYB58* (Figure 8). The transcriptional activation activities of *CgNAC043* and *CgMYB46* were analyzed using the yeast two-hybrid system—*CgNAC043* showed transcriptional activation activity in Y2H gold yeast and the activation motif was located in the C-terminal domain (Figure 7). In this network, *CgNAC043* may act as the master switch that activates the expression of many lignin biosynthetic genes.

In order to examine whether *CgNAC043* interacts with the promoters of lignin biosynthetic genes, we isolated the promoters of the *CgMYB46*, *CgC3H*, *CgCCoAOMT*, *Cg4CL*, and *CgCCR* genes from pomelo juice sacs. Our results indicated that *CgNAC043* not only activated the promoter of *CgMYB46*, but could also activate the promoter of *CgC3H* and *CgCCoAOMT* directly in vitro (Figure 9b), which is consistent with the regulation mode of *SND1-MYB46* lignin synthesis in *Arabidopsis*. Zhong [42] reported the consensus *SND1* binding sequence, (T/A)NN(C/T)(T/C/G)TNNNNNNNA(A/C)GN(A/C/T)(A/T), and designated this 19-bp imperfect palidromic sequence as the SWN binding element (SNBE). *SND1* together with other SWNs, including *VND6*, *VND7*, *NST1*, and *NST2*, bind to SNBE, designated as a secondary wall NAC binding element in the promoters of their direct targets. Genome-wide analysis of *SND1* and *VND7* revealed that they directly activate the expression of not only downstream TFs, but also a number of genes involved in secondary wall biosynthesis [42]. It was found that SNBEs are present in the promoters of all target genes of *CgMYB46*, *CgC3H*, and *CgCCoAOMT* (Table 1). So, we speculated that *CgNAC043* regulated juice sac granulation by recognizing the SNBE element of the gene promoter in the lignin synthesis pathway. Therefore, we believe that *CgNAC043* plays a very important role in the granulation of pomelo. Further study is need to determine how *CgNAC043* regulates juice sac granulation. In addition, our findings support the hypothesis that SWNs mediated the transcription regulation mechanism are conserved across others plants [49].

## 4. Materials and Methods

### 4.1. Plant Materials 

The San hong used in this study was grown in the FAFU experimental base in Fuqing City. To better understand the expression of *CgNAC043* at different developing stages and parts of fruits juice sacs, we collected samples at 157, 164, 172, 180, 188, 196, 204, and 212 days post anthesis (DPA). Every ninth fruit was picked from three different healthy trees and divided randomly into three replicates. The juice sacs from each sample were immediately separated, frozen in liquid nitrogen, and then stored at −80 °C.

### 4.2. Observation of Cell Walls of the Fruits Juice Sacs

The fruit juice sacs of San hong at 172 DPA and 212 DPA were sliced into thin sections. Thin sections were stained for lignin with a HCl−phloroglucinol solution [2% (*w*/*v*) phloroglucinol (dissolved in 95% (*v*/*v*) ethanol): 2M HCl = 1:1, fresh preparation] and observed under a Zeiss Axioscope A1 microscope (Leica) with a ×0.5 optical adapter. 

### 4.3. Gene Isolation

The full-length coding DNA sequence (CDS) of CgNAC043 (Cg5g040070) and the promoters of CgMYB46 (Cg2g003450), CgCCoAOMT (Cg8g004310), CgC3H (Cg6g017470), Cg4CL (Cg4g022000), and CgCCR (Cg2g003700) were download from the Citrus genome website (http://citrus.hzau.edu.cn/orange/download/index.php, accessed on 30 May 2020). The protein sequences were analyzed using BLAST at the NCBI database (https://blast.ncbi.nlm.nih.gov/Blast.cgi, accessed on 9 August 2021) to identify homologous sequences in other plant species.

The total RNA was isolated from pomelo fruit juice sacs using an RNA prep pure kit (BioFlux Biotech, Hangzhou, China). cDNA was synthesized with Transcript ^®^ One-step gDNA Removal and cDNA Synthesis SuperMix (TransGen, Beijing, China). The DNA was extracted using the CTAB method. The PCR amplification procedure was based on Phanta^®^ Max Super-Fidelity DNA Polymerase (Vazyme, Nanjing, China) and the vector construction on ClonExpressII One Step Cloning Kit (Vazyme, Nanjing, China). The primers used in this study are listed in Supplementary Table S2.

### 4.4. Bioinformatics Analysis

The amino acid sequences of the SWN genes were aligned using DNAMAN (8.0). The phylogenetic relationship of the second cell wall NAC proteins was constructed using MEGA7 software with 1000 bootstrap replications. The conserved domains of the NAC proteins were predicted used CD-search (https://www.ncbi.nlm.nih.gov/Structure/cdd/wrpsb.cgi, accessed on 4 December 2021). The conserved motif distribution was analyzed using the MEME Suite, and 10 motifs were identified (Figure 2b). The basic physical and chemical properties of the CDS sequence of *CgNAC043* were predicted by EXPASY (http://web.expasy.org/protparam, accessed on 20 December 2021 ).

### 4.5. Construction of Interaction Networks of CgNAC043 Protein in Pomelo

STRING (https://string-db.org/, accessed on 20 December 2021) was used to construct the functional protein association networks for the *CgNAC043* protein on the basis of *Arabidopsis* orthologs. The minimum required interaction score was set to medium confidence (0.400).

### 4.6. Transcriptional Activity Assay in Yeast

For the transactivation activity assays, the full-length C- and N-terminal sequences of *CgNAC043* and *CgMYB46* were amplified and inserted into the vector PGBKT7 (BD) at the NdeI and SmaI sites. The recombinant plasmids were then transformed into the yeast strain Y2H gold according to the manufacturer’s instructions, and the transformed yeast cells were grown on SD/–Trp (growth control), SD/–TDO, and SD/–TDO (+X-a-gal) plates to check the transactivation activity. The primers are listed in Supplementary Table S3.

### 4.7. RNA Extraction and Real-Time PCR Analysis

The total RNA was extracted from the juice sacs of different tissues at different stages using an RNA prep pure Kit (BioFlux Biotech, Hangzhou China). First-strand cDNA was synthesized using the PrimeScript RT Reagent Kit (TaKaRa, Dalian, China). For relative transcript level analysis, we used a SYBR^®^ Premix Ex TaqTM (Tli RNaseH Plus) (Takara, Dalian, China) and amplification was performed using the Jena qTOWER 2.2 real-time quantitative fluorescent PCR instrument. The normalized expression level of the gene in the tissues near the core at 157 DPA was used as a control value (expression set to 1), and the *β*-tublin gene was used as a reference gene for gene expression normalization [50]. All gene expression analyses were repeated using three biological replicates. The relative expression levels were calculated using the 2^−∆∆Ct^ method [51]. The primers are listed in Supplementary Table S4.

### 4.8. Transient Expression in Pomelo Mesocarp

Primers were designed based on the vector sequence (p1301), gene sequence, and enzyme cutting sites to construct the overexpression vector, p1301-*CgNAC043*. The empty p1301 and p1301-*CgNAC043* were transformed into *Agrobacterium* GV3101, then suspended in an infiltration buffer (10 mM MES, 10 mM MgCl_2_, 150 μM acetosyringone, pH 5.6), and the OD_600_ adjusted to 0.75. The p1301-*CgNAC043* was infiltrated on one side pericarp of San hong, and p1301 was infiltrated on the other side in the same fruit as the control (SK). Three days after infiltration, the treated tissues were used for the gene transient expression analysis. Three individual fruit replicates were used and the gene expression was analyzed by the 2^−ΔΔCT^ method. Gus stained referred to the X-gluc kit (Real-Times Biotechnology, Beijing, China), captured by the microscope (Leica).

### 4.9. Dual Luciferase Assay

Dual-luciferase assay is widely used for luciferase activity analysis [34,52,53]. The full-length sequence of *CgNAC043* was integrated into the pGreen II 0029 62-SK vector (effector). The promoters of *CgMYB46*, *CgC3H*, *CgCCoAOMT*, *Cg4CL*, and *CgCCR* were inserted into the pGreen II 0800-LUC vector (Reporter). All constructs were transfected into *Agrobacterium* GV3101, then resuspended in an infiltration buffer to an optimal density (OD_600_ = 0.75). Then, 1 mL of *Agrobacterium* cultures containing transcription factors were mixed with 250 µL of *Agrobacterium* containing promoters and the mixtures were injected into the leaves of *Nicotiana* *benthamiana* with needleless syringes. The pGreen II 0029 62-SK empty vector plus promoter was used as a control (set as 1). Three days after infiltration, the LUC and REN fluorescence intensities were measured using a Dual Luciferase Reporter Gene Assay Kit (Yeasen, Shanghai, China). The analysis was carried out with three replicates for each plant. The primers are listed in Supplementary Table S5.

## 5. Conclusions

In this study, *CgNAC043* was functionally analyzed, and it acts as a positive regulator of pomelo fruit lignification. *CgNAC043* regulates the biosynthesis of lignin by activating the transcription of *CgMYB46* and the lignin biosynthesis genes (*CgC3H* and *CgCCoAOMT*). We confirmed that *CgNAC043* had a similar function to *AtNST1* for secondary wall synthesis in *Arabidopsis*. Our findings support the hypothesis that the SWN-mediated transcription regulation mechanism is conserved across others plants.

## Figures and Tables

**Table 1:** Position of SNBE elements in the promoters. The number shown at the left of each sequence is the position of the first nucleotide relative to the start codon. The critical nucleotides in the SNBE sequences are shaded.

SNBE Element Position (Forward/Reverse)	Base Distribution																		
CgMYB46-SNBE1 -1253+	T	G	A	T	T	T	C	T	T	T	T	A	A	A	C	G	A	A	A
CgMYB46-SNBE2 -479+	T	T	A	T	G	T	G	A	A	G	T	G	G	A	A	G	C	A	A
CgMYB46-SNBE3 -372+	T	A	C	C	T	T	G	T	A	A	A	T	G	A	A	G	A	A	A
CgMYB46-SNBE4 -372-	T	T	T	C	T	T	C	A	T	T	T	A	C	A	A	G	G	T	A
CgMYB46-SNBE5 -454-	T	T	G	T	T	T	A	G	T	A	A	C	C	A	A	G	C	T	A
CgMYB46-SNBE6 -566-	A	C	A	C	G	T	G	T	A	T	G	T	C	A	A	G	A	T	A
CgC3H-SNBE1 -1412+	T	T	A	C	C	T	A	A	C	A	T	C	T	A	C	G	C	T	T
CgC3H-SNBE2 -1378+	T	A	G	C	T	T	A	A	G	A	A	A	G	A	A	G	G	C	A
CgC3H-SNBE3 -142+	T	A	A	T	T	T	C	T	T	A	A	C	A	A	C	G	T	A	A
CgC3H-SNBE4 -34+	A	A	G	T	T	T	C	A	A	G	A	A	A	A	A	G	G	A	A
CgC3H-SNBE -1378-	T	G	C	C	T	T	C	T	T	T	C	T	T	A	A	G	C	T	A
CgCCoAOMT1-SNBE1 -974+	A	T	A	T	G	T	G	A	G	C	G	T	G	A	A	G	A	C	T
CgCCoAOMT1-SNBE2 -651+	A	C	T	C	T	T	A	T	T	T	G	T	C	A	A	G	A	A	A
CgCCoAOMT1-SNBE3 -1734-	A	T	A	T	C	T	A	A	T	C	A	T	A	A	C	G	T	T	T
SNBE	T	N	N	C	T	T	N	N	N	N	N	N	N	A	A	G	N	A	A
consensus	A			T	C										C			C	T
					G													T	

## Data Availability

The data presented in this study are available in the article and Supplementary Materials.

## Supplementary Materials

The following supporting information can be downloaded at: https://www.mdpi.com/article/10.3390/plants11030403/s1. Supplementary Figure S1: Protein sequence alignment of *CgNAC043* with homolgs from others species. Supplementary Table S1: The FPKM values of *CgNACs* and *Cg**MYBs* in different tissues and different developmental stages of “San hong” were obtained from RNA-seq data of our lab. Supplementary Table S2: List of Primers used for gene cloning. Supplementary Table S3: List of Primers used for transcriptional activity assay. Supplementary Table S4: List of Primers used for RT-qPCR. Supplementary Table S5: List of Primers used for dual luciferace assays.

## References

[1] Shomer I., Chalutz E., Vasiliver R., Lomaniec E., Berman M. (1989). Scierification of juice sacs in pummelo (*Citrus grandis*) fruit. Can. J. Bot..

[2] Jia N., Liu J., Sun Y., Tan P., Cao H., Xie Y., Wen B., Gu T., Liu J., Li M. (2018). *Citrus sinensis* MYB transcription factors *CsMYB330* and *CsMYB308* regulate fruit juice sac lignification through fine-tuning expression of the *Cs4CL1* gene. Plant Sci..

[3] Zhang J., Wang M., Cheng F.S., Dai C., Sun Y.F., Lu J., Huang Y.T., Li M.M., He Y., Wang F.Z. (2016). Identification of microRNAs correlated with citrus granulation based on bioinformatics and molecular biology analysis. Postharvest Biol. Technol..

[4] Sharma R.R., Singh R., Saxena S.K. (2006). Characteristics of citrus fruits in relation to granulation. Sci. Hortic..

[5] Vanholme R., Demedts B., Morreel K., Ralph J., Boerjan W. (2010). Lignin biosynthesis and structure. Plant Physiol..

[6] Wu J., Pan T., Guo Z., Pan D. (2014). Specific lignin accumulation in granulated juice sacs of *Citrus maxima*. Agr. Food Chem..

[7] Olsen A.N., Ernst H.A., Leggio L.L., Skriver K. (2005). Nac transcription factors: Structurally distinct, functionally diverse. Trends Plant Sci..

[8] Aida M., Ishida T., Fukaki H., Tasaka F.M. (1997). Genes involved in organ separation in *A**rabidopsis*: An analysis of the cup-shaped cotyledon mutant. Plant Cell.

[9] Souer E., Van H.A., Kloos D., Mol J., Koes R. (1996). The no apical meristem gene of *Petunia* is required for pattern formation in embryos and flowers and is expressed at meristem and primordia boundaries. Cell.

[10] Zhong R., Ye D. (2006). *SND1*, a NAC domain transcription factor, is a key regulator of secondary wall synthesis in fibers of *Arabidopsis*. Plant Cell.

[11] Golfier P., Volkert C., He F., Rausch T., Wolf S. (2017). Regulation of secondary cell wall biosynthesis by a NAC transcription factor from miscanthus. Plant Direct.

[12] Taichi K., Naoki Y., Yuki T., Masaomi Y., Shiro S., Takefumi H., Mai M., Soichiro N., Daisuke S., Masahiro S. (2017). MYB-mediated upregulation of lignin biosynthesis in *Oryza sativa* towards biomass refinery. Plant Biotechnol. J..

[13] Takada S., Hibara K.I., Ishida T., Tasaka M. (2001). The CUP-SHAPED COTYLEDON1 gene of *Arabidopsis* regulates shoot apical meristem formation. Development.

[14] Vroemen C.W., Mordhorst A.P., Albrecht C., Kwaaitaal M.A., de Vries S.C. (2003). The CUP-SHAPED COTYLEDON3 gene is required for boundary and shoot meristem formation in *Arabidopsis*. Plant Cell.

[15] Yamaguchi M., Ohtani M., Mitsuda N., Kubo M., Ohme-Takagi M., Fukuda H., Demura T. (2010). VND-INTERACTING2, a NAC domain transcription factor, negatively regulates xylem vessel formation in *Arabidopsis*. Plant Cell.

[16] Xie Q., Guo H.S., Dallman G., Fang S., Weissman A.M., Chua N.H. (2002). *SINAT5* promotes ubiquitin-related degradation of *NAC1* to attenuate auxin signals. Nature.

[17] Kim Y.S., Kim S.G., Park J.E., Park H.Y., Lim M.H., Chua N.H., Park C.M. (2006). A membrane-bound NAC transcription factor regulates cell division in *Arabidopsis*. Plant Cell.

[18] Jensen M.K., Hagedorn P.H., De Torres-Zabala M., Grant M.R., Lyngkjaer M.F. (2010). Transcriptional regulation by an NAC (NAM-ATAF1,2-CUC2) transcription factor attenuates ABA signalling for efficient basal defence towards *Blumeria graminis* f. sp. hordei in Arabidopsis. Plant J..

[19] Wei S., Kuang J.F., Chen L., Xie H., Peng H.H., Xiao Y.Y., Li X.P., Chen W.X., He Q.G., Chen J.Y. (2012). Molecular characterization of banana NAC transcription factors and their interactions with ethylene signalling component EIL during fruit ripening. J. Exp. Bot..

[20] Ríos P., Argyris J., Vegas J., Leida C., Kenigswald M., Tzuri G., Troadec C., Bendahmane A., Katzir N., Picó B. (2017). *Ethqv6.3* is involved in melon climacteric fruit ripening and is encoded by a NAC domain transcription factor. Plant J..

[21] Nakashima K., Tran L., Dong V.N., Fujita M., Yamaguchi-Shinozaki K. (2010). Functional analysis of a NAC-type transcription factor osnac6 involved in abiotic and biotic stress-responsive gene expression in rice. Plant J..

[22] Nakashima K., Takasaki H., Mizoi J., Shinozaki K., Yamaguchi-Shinozaki K. (2012). NAC transcription factors in plant abiotic stress responses. Biochim. Biophys. Acta Gene Regul. Mech..

[23] Huang Q., Wang Y., Li B., Chang J.L., Chen M.J., Li K.X., Yang G.X., He G.Y. (2015). *TaNAC29*, a NAC transcription factor from wheat, enhances salt and drought tolerance in transgenic *Arabidopsis*. BMC Plant Biol..

[24] Yan H., Zhang A., Ye Y., Xu B., Chen J., He X., Wang C., Zhou S., Zhang X., Peng Y. (2017). Genome-wide survey of switchgrass NACs family provides new insights into motif and structure arrangements and reveals stress-related and tissue-specific *NACs*. Sci. Rep..

[25] Lee S., Seo P.J., Lee H.J., Park C.M. (2012). A NAC transcription factor *NTL4* promotes reactive oxygen species production during drought-induced leaf senescence in *Arabidopsis*. Plant J..

[26] Fan K., Bibi N., Gan S., Li F., Yuan S., Ni M., Wang M., Shen H., Wang X. (2015). A novel nap member *GhNAP* is involved in leaf senescence in *Gossypium hirsutum*. J. Exp. Bot..

[27] Mao C., Lu S., Bo L., Zhang B., Feng M. (2017). A rice NAC transcription factor promotes leaf senescence via ABA biosynthesis. Plant Physiol..

[28] Nobutaka M., Akira I., Hiroyuki Y., Masato Y., Motoaki S., Kazuo S., Masaru O.T. (2007). NAC transcription factors, *NST1* and *NST3*, are key regulators of the formation of secondary walls in woody tissues of *Arabidopsis*. Plant Cell.

[29] Mitsuda N., Ohme-Takagi M. (2008). NAC transcription factors *NST1* and *NST3* regulate pod shattering in a partially redundant manner by promoting secondary wall formation after the establishment of tissue identity. Plant J..

[30] Zhong R., Lee C., Zhou J., Mccarthy R.L., Ye Z.H. (2008). A battery of transcription factors involved in the regulation of secondary cell wall biosynthesis in *Arabidopsis*. Plant Cell.

[31] Li Q., Lin Y.C., Sun Y.H., Song J., Hao C., Zhang X.H., Sederoff R.R., Chiang V.L. (2012). Splice variant of the *SND1* transcription factor is a dominant negative of *SND1* members and their regulation in *Populus trichocarpa*. Proc. Natl Acad. Sci. USA.

[32] Chai M., Bellizzi M., Wan C., Cui Z., Wang G.L. (2015). The NAC transcription factor *OsSWN1* regulates secondary cell wall development in *Oryza sativa*. J. Plant Biol..

[33] Jie Z., Geng Q.H., Dan Z., Jing Q.Y., Yang L., Shan H., Xue B.L. (2018). The cotton (*Gossypium hirsutum*) NAC transcription factor (*FSN1*) as a positive regulator participates in controlling secondary cell wall biosynthesis and modification of fibers. New Phytol..

[34] Xu Q., Wang W., Zeng J., Zhang J., Grierson D., Li X., Yin X., Chen K. (2015). A NAC transcription factor, *EjNAC1*, affects lignification of loquat fruit by regulating lignin. Postharvest Biol. Technol..

[35] Hu P., Zhang K., Yang C. (2019). *BpNAC012* positively regulates abiotic stress responses and secondary wall biosynthesis. Plant Physiol..

[36] Zhong R., Richardson E.A., Ye Z.-H. (2007). The *MYB46* transcription factor is a direct target of *SND1* and regulates secondary wall biosynthesis in *Arabidopsis*. Plant Cell.

[37] McCarthy R.L., Zhong R., Ye Z.-H. (2009). *MYB83* is a direct target of *SND1* and acts redundantly with *MYB46* in the regulation of secondary cell wall biosynthesis in *Arabidopsis*. Plant Cell Physiol..

[38] Ko J., Kim W.C., Han K.H. (2009). Ectopic expression of *MYB46* identifies transcriptional regulatory genes involved in secondary wall biosynthesis in *Arabidopsis*. Plant J..

[39] Jia N., Liu J., Tan P.H., Sun Y.F., Lv Y.M., Liu J.M., Sun J., Huang Y.T., Lu J., Jin N. (2019). *Citrus sinensis* MYB transcription factor *CsMYB85* induce fruit juice sac lignification through interaction with other *CsMYB* transcription factors. Front. Plant Sci..

[40] Shi M., Liu X., Zhang H., He Z., Yang H., Chen J., Feng J., Yang W., Jiang Y., Yao J.L. (2020). The IAA- and ABA-responsive transcription factor *CgMYB58* upregulates lignin biosynthesis and triggers juice sac granulation in pummelo. Hortic. Res..

[41] Xu Q., Yin X.R., Zeng J.K., Ge H., Song M., Xu C.J., Li X., Ferguson I.B., Chen K.S. (2014). Activator- and repressor-type MYB transcription factors are involved in chilling injury induced flesh lignification in loquat via their interactions with the phenylpropanoid pathway. J. Exp. Bot..

[42] Zhong R., Lee C., Ye Z.H. (2010). Global analysis of direct targets of secondary wall NAC master switches in *Arabidopsis*. Mol. Plant.

[43] Zhong R.Q., Lee C.H., McCarthy R.L., Reeves C.K., Jones E.G., Ye Z.-H. (2011). Transcriptional activation of secondary wall biosynthesis by rice and maize NAC and MYB transcription factors. Plant Cell Physiol..

[44] Pan D., Zheng G., Chen G., She W., Guo Z., Shi M., Huiying A.L. (1999). Analysis of the reasons caused granulation of juice sacs in Guanximiyou pomelo variety. J. Fruit Sci..

[45] Zhong R.Q., Lee C.H., Ye Z.-H. (2010). Functional characterization of poplar wood-associated NAC domain transcription factors. Plant Physiol..

[46] Zhong R., Richardson E.A., Ye Z.-H. (2007). Two NAC domain transcription factors, *SND*1 and *NST1*, function redundantly in regulation of secondary wall synthesis in fibers of *Arabidopsis*. Planta.

[47] Hussey S.G., Mizrachi E., Spokevicius A.V., Bossinger G., Berger D.K., Myburg A.A. (2011). *SND2*, a NAC transcription factor gene, regulates genes involved in secondary cell wall development in *Arabidopsis* fibres and increases fibre cell area in *Eucalyptus*. BMC Plant Biol..

[48] Zhou J., Lee C., Zhong R., Ye Z.-H. (2009). *MYB58* and *MYB63* are transcriptional activators of the lignin biosynthetic pathway during secondary cell wall formation in *Arabidopsis*. Plant Cell.

[49] Zhong R., Lee C., Ye Z.-H. (2010). Evolutionary conservation of the transcriptional network regulating secondary cell wall biosynthesis. Trends Plant Sci..

[50] Xu Y. (2014). Cloning and Expression of Lignin Genes in Citrus maxima (Burm.) Merr[D].

[51] Pfaffl M.W. (2001). A new mathematical model for relative quantification in real-time RT-PCR. Nucleic Acids Res..

[52] Hellens R.P., Allan A.C., Friel E.N., Bolitho K., Grafton K., Templeton M.D., Karunairetnam S., Gleave A.P., Laing W.A. (2005). Transient expression vectors for functional genomics, quantification of promoter activity and RNA silencing in plants. Plant Methods.

[53] Min T., Fang F., Ge H., Shi Y.N., Luo Z.R., Yao Y.C., Donald G., Yin X.R., Chen K.S., Zhang J.S. (2014). Two novel anoxia-induced ethylene response factors that interact with promoters of deastringency-related genes from persimmon. PLoS ONE.

