# Supplementary material for: Identification and Functional Analysis of the CgNAC043 Gene Involved in Lignin Synthesis from Citrus grandis “San Hong”
Source: Plants (Basel). 2022 Jan 31;11(3):403. doi: 10.3390/plants11030403 (PMC8838788; doi:10.3390/plants11030403)
Supplement: Supplementary file 1 [file plants-11-00403-s001.zip › plants-1551142-supplementary.pdf]

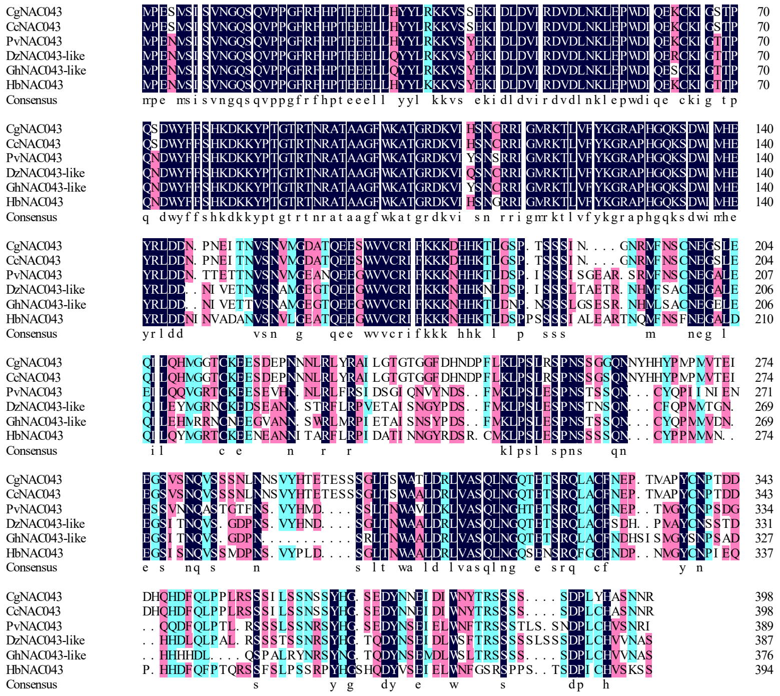

Supplementary Figure S1. Protein sequence alignment of *CgNAC043* with homolgs from *Citrus Clementina(Cc), Pistacia Vera(Pv), Durio zibethinus(Dz), Gossypium hirsutum(Gh)* and *Hevea brasiliensis(Hb).*

Supplemental Table S1. The FPKM values of *CgNACs* in different tissues and different developmental stages of ‘San hong’ were obtained from RNA-seq data of our lab.

id	157DPA-N	180DPA-N	212DPA-N	157DPA-F	180DPA-F	212DPA-F	Symbol	Description
Cg2g008130	0.293333333	4.01	8.13	0.04	1.006666667	2.796666667	NAC073	NAC domain-containing protein 73 [Citrus sinensis]
Cg5g040070	0.536666667	2.333333333	3.08	0.08	0.76	0.966666667	NAC043	NAC domain-containing protein 43 [Citrus clementina]
Cg9g019870	2.056666667	12.00333333	1.733333333	6.813333333	15.71666667	4.966666667	NAC062	NAC domain-containing protein 53-like [Citrus sinensis]
Cg2g042690	0.5	3.27	8.44	0.04	1.413333333	3.806666667	NAC073	NAC domain-containing protein 73 [Citrus sinensis]
Cg9g020010	3.116666667	15.70666667	4.053333333	8.23	22.61666667	6.873333333	NAC090	NAC domain-containing protein 53-like [Citrus sinensis]
Cg5g039810	10.14333333	21	20.09666667	11.58666667	27.15333333	25.38	NAC045	NAC domain-containing protein 16-like isoform X3 [Citrus sinensis]
Cg9g012980	1.483333333	4.99	2.116666667	4.093333333	7.526666667	1.08	NAC090	NAC domain-containing protein 53-like [Citrus sinensis]
CgUng000320	6.423333333	2.633333333	2.856666667	4.573333333	2.05	3.466666667	NAC071	NAC domain-containing protein 71 [Citrus clementina]
Cg3g022340	2.65	2.083333333	6.66	2.67	2.703333333	6.846666667	NAC014	NAC transcription factor 29-like isoform X2 [Citrus sinensis]
Cg3g025660	8.306666667	16.60333333	4.463333333	16.72	22.87	8.35	NAC031	protein CUP-SHAPED COTYLEDON 3 [Citrus clementina]
Cg6g025130	31.70333333	27.62666667	66.21666667	24.40666667	18.21666667	45.22333333	NAC100	NAC domain-containing protein 100 [Citrus clementina]
Cg5g000340	0	0.063333333	0.893333333	0	0	0.3	NAC012	NAC domain-containing protein 12-like [Citrus sinensis]
Cg5g033840	2.433333333	1.056666667	3.216666667	0.983333333	0.246666667	1.61	NAC021	NAC domain-containing protein 21/22 [Citrus clementina]
Cg5g010410	30.67333333	55.63666667	74.36	32.63333333	60.20666667	54.26333333	NAC100	NAC domain-containing protein 100 [Citrus sinensis]
Cg1g002260	0.153333333	0.346666667	1.223333333	0.036666667	0.24	0.6	NAC037	NAC domain-containing protein 76 [Citrus clementina]
Cg5g030590	8.35	13.35	18.41666667	12.58333333	22.98	15.03333333	NAC087	NAC domain-containing protein 87 [Citrus clementina]
Cg2g010310	2.763333333	6.266666667	6.976666667	5.423333333	7.59	6.863333333	NAC083	NAC domain-containing protein 83 [Citrus clementina]
Cg9g012960	1.036666667	1.906666667	0.323333333	0.82	4.78	2.016666667	NAC090	NAC domain-containing protein 53-like [Citrus sinensis]
Cg9g029500	2.603333333	1.693333333	1.416666667	2.046666667	1.15	0.663333333	NAC086	NAC domain-containing protein 45 [Citrus clementina]

Note: N: The tissues of near the core; F: The tissues of far away from the core.

Supplemental Table S1. The FPKM values of *CgMYBs* in different tissues and different developmental stages of 'San hong' were obtained from RNA-seq data of our lab (continued).

	157DPA-N	180DPA-N	212DPA-N	157DPA-F	180DPA-F	212DPA-F	Symbol	Description
Cg5g013190	12.18	19.27666667	34.51	2.826666667	6.96	14.81333333	MYB61	MYB61, partial [Citrus sinensis]
Cg9g028980	1.993333333	4.8	21.75666667	0.24	1.786666667	7.876666667	MYB58	transcription factor MYB58 [Citrus sinensis]
Cg6g008620	47.11666667	32.63333333	26.43	22.35333333	15.08666667	16.78333333	MYB60	myb-related protein 306 [Citrus clementina]
Cg9g001630	4.86	0.683333333	2.846666667	5.553333333	1.013333333	3.643333333	MYB111	MYB transcription factor [Citrus sinensis]
Cg5g002230	0.323333333	3.55	5.62	0.023333333	1.04	2.59	MYB61	transcription factor MYB10 [Citrus sinensis]
Cg1g013930	18.19666667	43.28666667	25.49666667	29.87666667	47.22333333	28.43333333	MYBS3	transcription factor MYBS3 [Citrus sinensis]
Cg6g025140	4.23	1.236666667	1.923333333	4.19	1.27	2.01	MYB16	transcription factor MYB16 [Citrus clementina]
Cg2g041090	0.996666667	5.906666667	1.216666667	1.843333333	5.013333333	1.47	MYB4	myb-related protein 308-like isoform X1 [Citrus sinensis]
Cg5g042300	11.32333333	5.33	4.536666667	8.236666667	4.616666667	5.523333333	MYB17	transcription factor MYB41 [Citrus clementina]
Cg2g021930	0.056666667	0.573333333	0.51	0.29	0.326666667	0.516666667	MYB102	transcription factor MYB41 [Citrus sinensis]
Cg6g018200	6.093333333	7.82	19.33	6.77	4.123333333	11.65	MYB20	transcription factor MYB20 [Citrus clementina]
Cg5g022560	2.816666667	2.67	7.54	2.07	2.05	4.21	MYB62	transcription factor MYB108-like [Citrus sinensis]
Cg3g019290	2.506666667	1.913333333	4.836666667	1.5	1.386666667	2.333333333	MYB330	transcription repressor MYB6-like [Citrus sinensis]
Cg1g008350	1.136666667	2.573333333	8.033333333	0.233333333	0.966666667	2.663333333	MYB78	transcription factor JAMYB [Citrus clementina]
Cg8g017210	0.03	0.293333333	1.77	0	0.086666667	0.583333333	MYB52	transcription factor MYB54 [Citrus clementina]
Cg3g017650	0.42	1.136666667	3.176666667	0.046666667	0.576666667	1.31	MYB52	transcription factor MYB115-like [Citrus sinensis]
Cg3g021810	81.64666667	46.12333333	25.67666667	105.5833333	59.35	51.69333333	MYB77	transcription factor MYB77 [Citrus clementina]
Cg2g003450	0.446666667	0.793333333	2.07	0	0.506666667	1.063333333	MYB46	transcription factor MYB46 [Citrus clementina]
Cg2g000510	7.74	5.84	4.2	11.24333333	9.146666667	4.923333333	MYB5	R2R3-MYB family transcription factor [Citrus aurantium]
Cg1g008900	4.02	4.976666667	7.24	2.493333333	3.19	5.59	MYB330	myb-related protein 308-like [Citrus sinensis]

Supplemental Table S2. Information for the primers used in this study

Primer Name	*Forward primer (5′ to 3′)*	*Reverse primer (5′ to 3′)*	Application
CgNAC043	ATGCCTGAAAGCATGAGTATATCAG	TTATAACCTGTTATTTGACGCGTG	
CgMYB46-Pro	GGCATGGCCACCAATTAT	TTTAAGAGACACACCTTTTGGATT	
CgProC3H-Pro	AAGTTAGGTC GCGGGAAGTG	CTCAGCCGTTGGTAGAGCTT	Gene cloning
CgCCR-Pro	CCTCCCAGTGGTCATGTAGC	CTGCTGCACCGCTCATTTTC	
Cg4CL-Pro	GGTCGGATTGGTTGCATAGC	AGGGAGGTGATTTGGGATGT	
CgCCOAOMT-Pro	AACGTCAGAGCAGTATAGGAGAG	TGACGCAAGTCTTTTCTTTTGAT	

Supplemental Table S3. Information for the primers used in this study

Primer Name	*Forward primer (5′ to 3′)*	*Reverse primer (5′ to 3′)*	Application
CgNAC043(1-42<br>9)	TCAGAGGAGGACCTGCATATGATG<br>CCTGAAAGCATGAGTATA	GCAGGTCGACGGATCCCCGGGGAGT<br>CTATAT TCATGCATGA	
CgNAC043(430-<br>1197)	TCAGAGGAGGACCTGCATATGGAC<br>GACAATCCCAATGAAATTAC	GCAGGTCGACGGATCCCCGGGTAAC<br>CTGTTATTTGACGCGTG	
CgNAC043(1-11<br>97)	TCAGAGGAGGACCTGCATATGATG<br>CCTGAAAGCATGAGTATA	GCAGGTCGACGGATCCCCGGGTAAC<br>CTGTTATTTGACGCGTG	Transcriptional activity assay
CgMYB46(1-378)	TCAGAGGAGGACCTGCATATGATG<br>AGAAAGCCGGAAAATAACG	GCAGGTCGACGGATCCCCGGGCTTG<br>AGCCTTTTCTTGATTG	
CgMYB46(379-9<br>48)	TCAGAGGAGGACCTGCATATGAAT<br>TTATCATCATCATCATCAACAC	GCAGGTCGACGGATCCCCGGGTGAC<br>CAACTTGAGTAGAAATCA	
CgMYB46(1-948)	TCAGAGGAGGACCTGCATATGATG<br>AGAAAGCCGGAAAATAACG	GCAGGTCGACGGATCCCCGGGTGAC<br>CAACTTGAGTAGAAATCA	

Supplemental Table S4. Information for the primers used in this study

Primer Name	*Forward primer (5′ to 3′)*	*Reverse primer (5′ to 3′)*	Application
CgNAC043	CGAACTCCTCTGGTAGCCAA	GATGATGACTCCGTCTCCGT	
CgMYB46	ATAACGGCAACGGTAACAGC	GCCCAGCATTTCTAGCAACA	
CgMYB58	CAGCCGAACATGGTTCCAAT	GGTCCACTTCTGGTTTCAGC	
CgC3H	CACGTCAACGTATGGGCAAT	GGCCTTTCATGTCCACATCC	qRT-PCR
CgCCoAOMT	CACAAGAGCCTGCTCCAATC	TCAAGAACTGCCCTTCGTCT	
Tublin	ACATCCCGCCTAAGGGTCTG	TTCCTCCGAAACATAGCCGTA	

Supplemental Table S5. Information for the primers used in this study.

Primer Name	*Forward primer (5′ to 3′)*	*Reverse primer (5′ to 3′)*	Application
62SK-CgNAC043	CGCTCTAGAACTAGTGGATCCATG<br>CCTGAAAGCATGAGTATATCAG	TCAGCGTACCGAATTGGTACCTTAT<br>AACCTGTTATTTGACGCGTGG	
LUC-CgMYB46-<br>Pro	CTATAGGGCGAATTGGGTACCGGC<br>ATGGCCACCAATTAT	TGTTTTTGGCGTCTTCCATGGTTTAA<br>GAGACACACCTTTTGGATAT	
LUC-CgProC3H-<br>Pro	CTATAGGGCGAATTGGGTACCTTT<br>CGGCTTATCCATTACCATTAGG	TGTTTTTGGCGTCTTCCATGGTTTGTG<br>TTGGTTGGTTTCCTTTTTC	Dual luciferace assay
LUC-CgCCR-Pro	CTATAGGGCGAATTGGGTACCACC<br>TATCACTACTTCTCTTATTCAC	TGTTTTTGGCGTCTTCCATGGTTTCA<br>GATACTGATTCTTCGTAATC	
LUC-Cg4CL-Pro	CTATAGGGCGAATTGGGTACCTTT<br>GTTATCTACCTTATCCTTGACT	TGTTTTTGGCGTCTTCCATGGTTTTGC<br>AAAGAGAATATGGATATG	
LUC-CgCCOAO<br>MT-Pro	CTATAGGGCGAATTGGGTACCAAC<br>GTCAGAGCAGTATAGGAGAG	TGTTTTTGGCGTCTTCCATGGTGACG<br>CAAGTCTTTTCTTTTGAT	
